# Nematode Autotomy Requires Molting and Entails Tissue Healing without Obvious Regeneration

**DOI:** 10.3390/jdb7040021

**Published:** 2019-11-23

**Authors:** Jonathan Hodgkin

**Affiliations:** Department of Biochemistry, University of Oxford, Oxford OX1 3QU, UK; jonathan.hodgkin@bioch.ox.ac.uk

**Keywords:** autotomy, nematode, *C. elegans*, molting, ecdysis, wound-healing, regeneration

## Abstract

Autotomy in *C. elegans*, which results in the severing of the body into two fragments, has been observed as a response to late larval worm-star formation after exposure to a bacterial surface pathogen. It was found that autotomy can occur in both hermaphroditic and gonochoristic nematode species, and during either the L3 or the L4 molt. Severing was hypothesized to be driven by a ‘balloon-twisting’ mechanism during molting but was found to be independent of lethargus-associated flipping. Extensive healing and apparent tissue fusion were seen at the site of scission. No obvious regeneration of lost body parts was seen in either L4 or adult truncated worms. A variety of mutants defective in processes of cell death, healing, regeneration, responses to damage, stress or pathogens were found to be competent to autotomize. Mutants specifically defective in autotomy have yet to be found. Autotomy may represent a modification of the essential normal process of molting.

## 1. Introduction

Autotomy, defined as the shedding of a body part, is a widespread phenomenon in the animal kingdom, often serving as a means of escape from entrapment or for removal of a damaged body part [1,2]. It usually requires mechanisms both for achieving shedding and for the consequent healing of the associated traumatic damage. Autotomy may also be followed by regeneration of the lost body part. It can therefore be viewed as a specialized modification of normal developmental processes. Despite its occurrence in many invertebrate taxa and in some vertebrates, such as various amphibia and reptiles, autotomy was believed to be absent from all members of the large invertebrate phylum Nematoda. These worms are characterized by having a hydrostatic skeleton, maintained by high internal turgor pressure, and consequently it would seem difficult for nematodes to undergo autotomy without exploding.

Recently [3], autotomy was observed in the nematode *Caenorhabditis elegans*, after larval animals had been induced to form ‘worm-stars’ by exposing swimming worms to the pathogenic bacterium *Leucobacter celer astrifaciens*, strain CBX151 (hereafter referred to as Verde1). This bacterium caused the tails of worms swimming in liquid to stick together and, ultimately, to form irreversible, star-like aggregates, with lethal consequences to the trapped worms. Larval worms, but not adults, could escape from these aggregates by undergoing a process of autotomy during the final larval molt, resulting in the formation of truncated adult animals that had lost between 5% and 90% of their bodies posterior to the pharynx. These truncated animals were still capable of movement and pharyngeal pumping, but tended to die after a few hours of feeding, as a result of lethal constipation due to a sealed-off intestinal lumen. Autotomized hermaphrodites that contained sufficient remnants of their gonads were able to produce progeny, thereby demonstrating the survival value of autotomy as a means of escape from Verde1 pathogenesis.

The occurrence of nematode autotomy is surprising and raises multiple questions about the mechanisms involved. How general is the phenomenon? How is body separation achieved? What healing processes occur in order to survive the massive trauma? Is autotomy followed by any regeneration? Are identifiable developmental signaling pathways involved? The analysis in this paper addresses some of these questions. It appears that autotomy can occur during molting in many rhabditid nematodes; that the body severing is achieved by a twisting process associated with ecdysis; and that tissue layers are sealed off without any obvious regeneration or proliferation in the damaged tissues. None of the many tested developmental regulators were found to be essential for autotomy.

## 2. Materials and Methods

### 2.1. C. elegans Growth Conditions

Standard conditions for growth and culture of *C. elegans* and other soil nematodes were used [4]. Carbenicillin to 25 μg/mL, chloramphenicol to 25 μg/mL, or aldicarb to 0.1 mm were added as appropriate.

### 2.2. Formation of Worm-Stars

Standard conditions for creating larval worm-stars and testing them for ability to undergo autotomy were established [3]. Strain CBX151 (Verde1) bacteria were grown to stationary phase in nutrient broth (LB or 2XTY) and diluted to 10^8^ cfu/mL in M9 buffer. This suspension was kept at 4 °C and remained capable of inducing worm-stars for at least 4 weeks. Worms to be tested were approximately synchronized by picking rafts of eggs from adult populations, and grown to L3 or L4 stage at 15 °C. A procedure similar to that for making adult worm-stars was used: synchronous populations of 200–2000 larvae were washed into 1 mL of M9 buffer in a microfuge tube, and allowed to settle for 10–15 min. A total of 100 μL of settled worms at the bottom of the tube were then transferred to the lid of a small plastic Petri dish, and 50 μL of Verde1 suspension was added to this drop. Worm-stars usually began to form in the drop within three minutes, and were allowed to mature and stabilize for a further 10–15 min. They were then sucked up in 10 μL volumes using a micropipette tip, washed for a few minutes by transfer to separate drops of M9 buffer, and then deposited on NGM agar plates, in the absence of a bacterial lawn. After incubation for 24 h at room temperature, plates were examined for the appearance of autotomized worms. This procedure worked efficiently for most of the tested strains; however, mutants that are unable to swim vigorously, such as many of those with an Unc (uncoordinated) phenotype, were unable to form worm-stars. Conversely, some mutants, especially those with abnormal surface properties, were hypersensitive to Verde1 and formed disordered tangles rather than stars when exposed to this pathogen, so conditions allowing autotomy were absent.

### 2.3. Strains

Bristol N2 was used as the reference wildtype strain. Other natural isolates of *C. elegans* were: AB1, CB4856, CX11262, JU311, JU1400, JU1652, LKC31, MY23, VC2010.

Other Caenorhabditis strains were: *C. brenneri* (CB5161); *C. briggsae* (AF16, DH1300, HK104, HK105, JU1038, PB826, DH1300); *C. doughertyi* (JU1333); *C. japonica* (DF5081); *C. nigoni* (JU1422); *C. portoensis* (EG4788); *C. remanei* (EM464, PB219); *C. sinica* (JU1201); *C. tropicalis* (CB6879). Other nematode strains were: *Oscheius tipulae* (CEW12), *Pristionchus pacificus* (PS312, PS1843), *Panagrellus redivivus* (MT8872).

Mutant genes and alleles examined were: *aff-1(tm2214)*, *aptf-1(gk794)*, *bus-10(e2702)*, *ced-1(e1735)*, *ced-2(e1752)*, *ced-3(n717)*, *ced-3(n1286)*, *ced-4(n1162)*, *ced-6(n2095)*, *ced-7(n1892)*, *ced-8(n1891)*, *ced-9(n1950gf)*, *ced-12(n3261)*, *ced-13(sv32)*, *ceh-10(ct78)*, *ceh-24(cc539)*, *ceh-24(tm1103)*, *csp-1(n4967)*, *csp-2(n4871)*, *csp-3(ok1563)*, *cwp-4(ok2548)*, *daf-16(mu86)*, *dapk-1(ju4)*, *dbl-1(wk70)*, *dcar-1(tm2484)*, *dlk-1(ju476)*, *dpy-11(e224)*, *dpy-17(e164)*, *efa-6(ok3533)*, *eff-1(hy21)*, *eff-1(oj55)*, *egl-1(n1084n3082)*, *egl-9(sa307)*, *fbn-1(ns67)*, *hif-1(ia4)*, *him-8(e1489)*, *hsf-1(sy441)*, *jkk-1(km2)*, *jnk-1(gk7)*, *kgb-2(gk361)*, *lim-9(gk109)*, *lin-2(e1309)*, *lin-4(e912)*, *lin-42(n1089)*, *lon-1(e185)*, *lon-2(e678)*, *lon-3(e2175)*, *mak-2(gk1110)*, *max-2(cy2)*, *mig-1(e1787)*, *mpk-1(ku1)*, *mkk-4(ok1545)*, *mlk-1(ok2471)*, *mtm-1(op309)*, *nlg-1(ok259)*, *nsy-1(ag3)*, *nuc-1(e1392)*, *pik-1(tm2167)*, *pmk-1(ku25)*, *pmk-3(ok169)*, *psr-1(ok714)*, *ptr-23(ok3663)*, *rbr-2(ok2544)*, *rict-1(ft7)*, *rol-6(su1006)*, *sdn-1(ok449)*, *sli-1(sy143)*, *sma-2(e502)*, *sod-1(tm776)*, *wrk-1(ok695)*.

### 2.4. Photography

Worm-stars and autotomized worms were anesthetized with 10 mm sodium azide and mounted on 2% agarose pads. Images were captured by differential interference contrast (*DIC*) microscopy on a Zeiss Standard 16 microscope using a Moticam 580 camera, or on a Zeiss Axioplan 2 fluorescence microscope using a Zeiss AxioCam camera.

## 3. Results

### 3.1. Autotomy Can Occur in Many Rhabditid Species

Conditions for permitting autotomy were explored: as previously reported [3], about 5% of L4 larval worms trapped in a worm-star were seen to undergo autotomy. Synchronous late L4 worms, grown at 15 °C and collected prior to lethargus, were found to be optimal material, such that up to 10% of trapped worms underwent autotomy. The process of autotomy did not depend on continued presence of the Verde1 bacteria. This was demonstrated by allowing L4 worm-stars to form by exposure to low concentrations of Verde1 (<10^7^ cfu/mL), and then incubating these worm-stars on NGM agar with added carbenicillin or chloramphenicol. Verde1 bacteria are sensitive to these antibiotics [5]. The worm-stars failed to disassemble under these conditions and generated autotomized adults with similar frequency to untreated worm-stars. Tail-knotting of larvae therefore appeared to be a sufficient condition for autotomy, implying that it is a mechanical consequence rather than a specific response to the pathogen.

**Figure 1:**
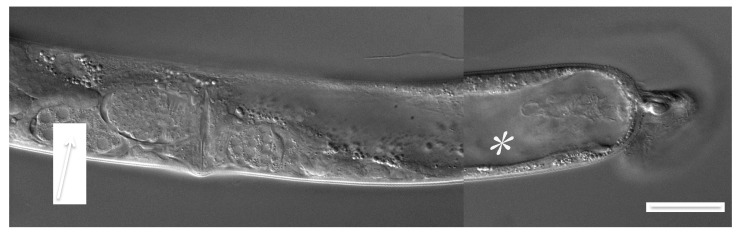
Posterior part of an autotomized and fertilized female of *C. remanei.* Note developing eggs (arrow) and blocked gut lumen (*). Scale bar 50 microns.

As previously reported [3], Verde1 can induce adult worm-star formation in most tested Caenorhabditis species, as well as in the related *Oscheius tipulae.* Late larval cultures of several of these species were tested for susceptibility to autotomy after worm-star formation. Most of those examined were able to form autotomized worms, although usually with lower frequency than was seen for *C. elegans.* Both hermaphroditic species (*C. briggsae*, *C. tropicalis*, *O. tipulae*) and gonochoristic species (*C. remanei*, *C. brenneri*, *C. nigoni*, *C. sinica*, *C. portoensis*, *C. doughertyi*) were seen to be capable of producing autotomized adults. Adult females of *C. remanei* and other gonochoristic species could be successfully fertilized by intact adult conspecific males after autotomy, even if they had lost some of their post-vulval anatomy (Figure 1), thereby demonstrating that female tails are not needed for successful mate recognition, copulation and reproduction. Multiple independently isolated races of both *C. elegans* and *C. briggsae* were tested for their ability to form worm-stars and autotomize, in order to explore natural variability in these traits. All ten tested *C. elegans* races exhibited similar competence, but two out of six *C. briggsae* races failed to form worm-stars (*PB826, DH1300*), indicating significant variability in susceptibility to Verde1 in this species.

Some of the examined species appeared unable to form larval worm-stars, with failure due to sluggish swimming (*C. japonica*), inefficient bacterial adhesion (*Pristionchus pacificus*) or lethal hypersensitivity to Verde1 (*Panagrellus redivivus*) [5], and therefore it was not established whether they can ever experience autotomy. Nevertheless, the results of this survey suggest that the ability to undergo autotomy may be widespread among small nematodes. Aggregates resembling worm-stars, described as Medusa-heads or rosettes, have been seen forming in populations of both parasitic and free-living nematodes [6,7], so it may be that some of these also may lead to autotomy in the trapped worms.

### 3.2. Autotomy Can Occur at Either L3-to-L4 or L4-to-Adult Molts

**Figure 2:**
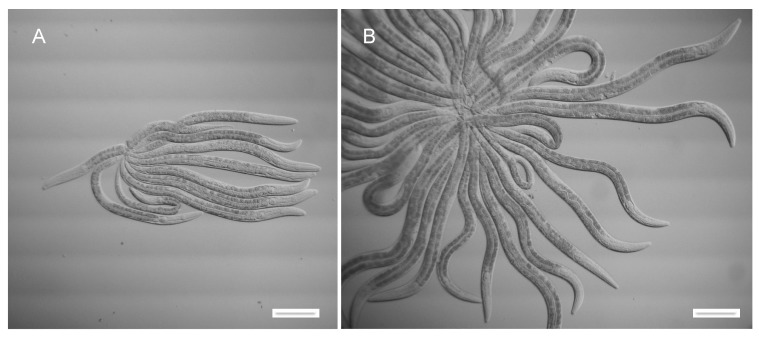
Larval worm stars. (**A**) L3 worm-star. (**B**) L4 worm-star. Scale bar 100 microns.

Autotomy was previously described as occurring at the L4-to-adult molt, after formation of L4 worm-stars (Figure 2B). Stars were also seen to form at earlier larval stages, albeit with lower efficiency, probably as a result of the shorter tail spikes and less vigorous swimming of worms at these stages. Synchronous populations of L3 worms were prepared and exposed to Verde1 bacteria in liquid at high density (>10^4^ worms/mL). Under these conditions, stable L3 worm-stars could be formed (Figure 2A).

**Figure 3:**
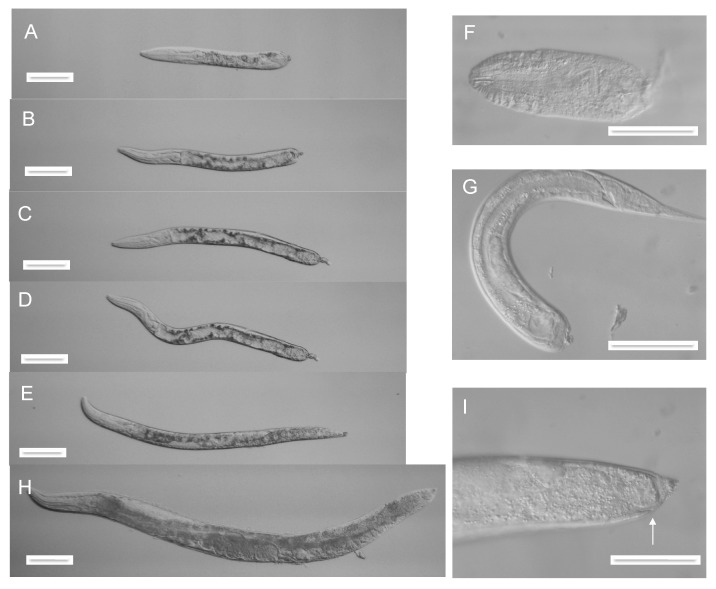
L4 stage autotomy. (**A**–**E**) Autotomized L4 larvae. (**F**) Autotomized L4 pharynx. (**G**) Autotomized L4 larval tail. (**H**). Adult derived from an L4 larva with post-anal autotomy. (I) Tail region of animal in (**H**), with anus arrowed. Scale bar 100 microns (**A**–**E**,**H**) or 50 microns (**F**–**I**).

When these L3 stars were plated on unseeded NGM agar, some of the trapped worms underwent autotomy during the L3-to-L4 molt, resulting in autotomized L4 larvae. As with the autotomized adults, scission appeared to occur at any point posterior to the pharynx (Figure 3A–F). Most of these worms continued to exhibit pharyngeal pumping and some body movement for at least 24 h after escape from the parent worm-star.

L4 worms were transferred to bacterial lawns and allowed to develop. Those that had undergone truncation anterior to the anus (Figure 3A–D) failed to reach the L4 molt and died with greatly distended intestinal lumina (22/22). In contrast, most of those with post-anal truncation (Figure 3E) were able to molt and reached fertile adulthood (9/10). However, these adults invariably lacked tail-spikes and other post-anal structures, indicating that no regeneration of these structures had occurred (Figure 3H,I).

It is possible that autotomy could also occur at the L1 and L2 molts, if stably knotted worm-stars could be established at these stages. Autotomy of adult worms has not been observed, perhaps because no further molting occurs, or because worms at this stage are too large or too rigid to permit autotomy. Certain heterochronic mutants, such as *lin-4*, undergo a fifth molt during adulthood [8] and might therefore be able to undergo adult autotomy. To test this possibility, young adult *lin-4* mutant worms were exposed to Verde1 bacteria in liquid, under standard star-inducing conditions. Unfortunately, these worms failed to swim vigorously and formed only unstable tangles rather than stars.

### 3.3. Autotomy Can Occur at Any Body Axis Point Posterior to the Pharynx

**Figure 4:**
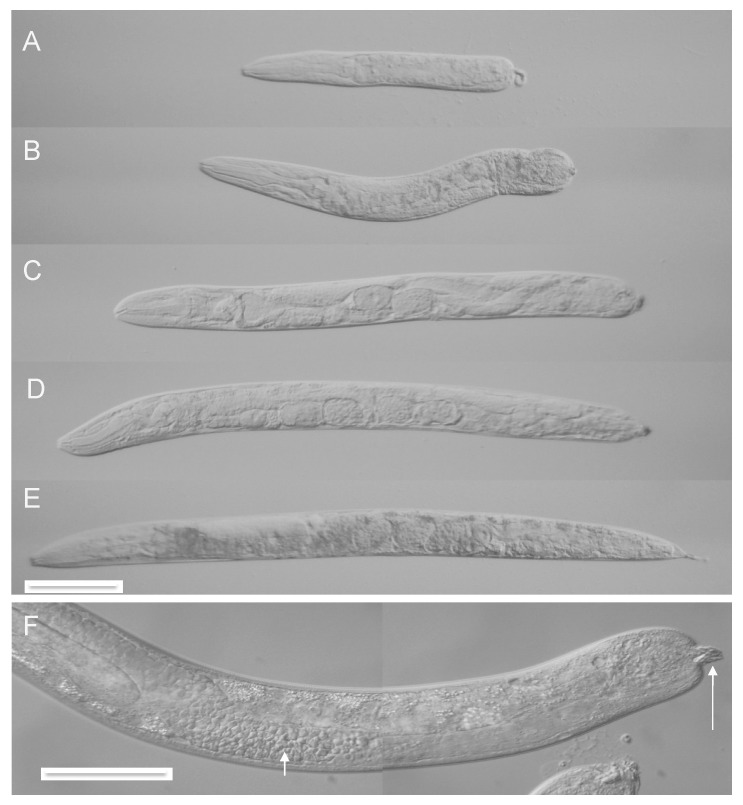
Adult autotomy. (**A**–**E**) Automized adult hermaphrodites. Scale bar 100 microns. (**F**) Autotomized adult male; note mature spermatids (short arrow) and spicule remnants at the site of truncation (long arrow).

Both L4 and adult autotomized worms of many different lengths were observed (Figure 3 and Figure 4), ranging from those that have undergone scission immediately behind the pharynx (Figure 3F), to those that have lost only the most posterior part of the tail spike (Figure 4E) Scission occurred most commonly in the posterior half of the body, but no obvious preferences in the site of scission have been seen, apart from avoidance of the pharynx, which presumably is too mechanically robust to allow scission. Also, it is possible that scission in the region around the developing vulva in a molting L4 worm may lead to lethal rupture, as a result of the mechanical weakness of the vulval opening.

Autotomy in the middle or anterior part of a worm might be expected to yield a surviving posterior fragment as well as the anterior fragment. Such fragments were very occasionally observed, such as the L4 tail fragment shown in Figure 3G. They usually retained a limited ability to move for at least 12 h after formation, indicating that the muscles and nerves in the isolated tail fragment remained functional. They exhibited sealed-off anterior ends, similar to the sealed-off posterior ends of head fragments.

### 3.4. Autotomy may be Achieved by Twisting

**Figure 5:**
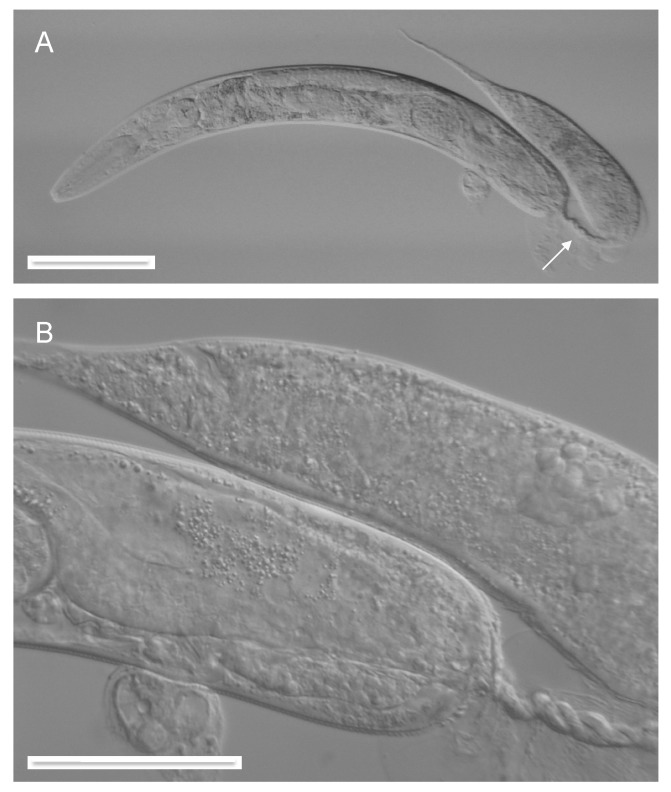
Autotomy by twisting. (**A**) Incompletely autotomized adult; arrow indicates residual link between fragments. Scale bar 100 microns. (**B**) Detail of twisted isthmus between fragments. Scale bar 50 microns.

Surviving autotomized worms frequently exhibited a twisted cuticular projection at their posterior end. This suggested that autotomy may have resulted from a twisting process, which constricted the internal organs of the worm down to a point where the body could be divided. Examination of incompletely autotomized adults, which were occasionally seen, provided evidence for this mechanism, because multiple twists were seen in the residual cuticular isthmus between anterior and posterior body fragments (Figure 5). On a much larger scale, a twisting procedure can be used to split up pressurized balloons, as in the familiar generation of ‘balloon animals’. For the worms, a comparable process should permit separation without rupture, despite the internal turgor of the animals.

Flipping or spinning along the longitudinal axis of the worm is a characteristic transient behavior that occurs during nematode ecdysis [9,10], which has been interpreted as assisting apolysis, i.e., the removal of old cuticle from the preceding larval stage. If the posterior end of a molting worm were to be immobilized in a worm-star, the flipping might provide enough mechanical force to twist the worm and create a constriction, followed by severance. Recent work on flipping during lethargus has defined the neuroanatomical basis of this behavior, and demonstrated that the homeobox gene *ceh-24* is required for the formation of the necessary processes and cholinergic function in the relevant SIA motor neurons [11]. Mutants of this gene are defective in flipping during lethargus but otherwise almost normal. Surprisingly, they exhibit no abnormality or delay in molting. Two deletion mutants of *ceh-24* and a mutant of the lethargus regulator *aptf-1* [11] were tested and found to be fully competent for autotomy, demonstrating that lethargus-associated flipping is not essential for autotomy. However, the worms carrying these mutations were still seen to be writhing around each other in three dimensions when trapped in worm-stars, so the twisting mechanism for autotomy remains plausible.

Evidence for the importance of movement in order to achieve autotomy was obtained by plating L4 worm-stars on NGM agar containing a sub-lethal concentration of aldicarb (0.1 mm), which severely impairs movement but allows molting and maturation to fertile adulthood. Under these conditions, autotomy was almost completely inhibited (ten aldicarb-treated L4 worm-stars yielded only two autotomized adults, as compared to at least 36 for 11 control L4 worm-stars). The treated worm-stars were gently teased apart in order to detect any additional autotomized worms, but none were found.

### 3.5. Autotomy is Associated with Extensive Healing and May Involve Tissue Fusions

Inspection at the level of light microscopy of autotomized animals, both L4 and adult, suggested that these animals had undergone tissue fusions or adhesions in both epidermal and endodermal layers, as part of the process of autotomy. They were able to move and maintain body shape and internal turgor, indicating a mechanically intact epidermis and a functioning hydrostatic skeleton. Also, their intestinal lumina became grossly distended without release of gut contents into the body cavity, indicating that the intestinal epithelia were also mechanically intact. Bloated gut lumina are visible in many of the images of autotomized words (Figure 1, Figure 3 and Figure 4).

Other tissues may also undergo apparent fusion, such as the male reproductive tract. Autotomized adult males were generated, though only rarely because L4 males were difficult to trap into worm-stars. Most truncated adult males had lost all of their posterior copulatory structures and were incapable of mating but many were seen to be undergoing spermatogenesis (Figure 4F), sometimes with mature spermatids accumulating in a testicular compartment, presumably a truncated seminal vesicle. Spermatids were not seen to escape into the main body cavity, indicating that the reproductive tract had been sealed off.

Extensive cell fusion events therefore seem likely, but it is conceivable that extracellular scar tissue or cell junctions, rather than cell fusions, may provide additional mechanical integrity in epidermis and intestine. Matrices and mucus produced over the course of the molt may also seal the wound in the absence of cell–cell fusion. Detailed cellular and sub-cellular description of the wound healing and remodeling at the site of autotomy would require examination by electron microscopy. This should also reveal what happens to the muscles and nerves in the region of scission.

Two genes specifically required for various cell fusions in *C. elegans* have been identified: *Eff-1* and *aff-1* [12]. Mutants of these genes were therefore examined, but both of the single mutants were found to be competent for autotomy. However, these genes are partly redundant in their action, and double *aff-1; eff-1* mutants are inviable, so it is possible that they are redundantly required for successful autotomy. The *eff-1* paralog *C26D10.7* (not tested) might also contribute.

### 3.6. Autotomy Fails to Induce Obvious Cell or Tissue Regeneration

As noted above, post-anal truncation of L4 worms failed to elicit re-growth of tail structures during later development (Figure 3H,I). Similarly, adult worms that had been truncated at the final molt were never seen to regenerate any of the missing structures. Adults that had undergone autotomy anterior to the anus usually died within 24 h of being allowed to feed, as a result of lethal constipation and eventual rupture, but in the absence of food they often survived and continued to move for several days. However, no signs of regeneration were seen in these animals. Adults with post-anal truncation survived longer (Figure 4D,E) but also failed to exhibit any detectable regeneration. Additional cell divisions after autotomy were not explicitly searched for but would have been difficult to detect.

### 3.7. Successful Autotomy is Independent of Most Signaling Pathways or Regeneration Mechanisms

A great deal is known about cell and tissue shaping and remodeling during *C. elegans* development, in a variety of contexts: embryonic and post-embryonic morphogenesis, cell migration and navigation, cell death and corpse clearance, neuronal regeneration. Any of the cellular mechanisms required for these processes might potentially also be necessary for autotomy, or alternatively to suppress it. Therefore, various mutant strains were tested for the ability to undergo autotomy. Necessarily, not all of the many thousands of available mutants of *C. elegans* have been tested. Also, subtle alterations in the efficiency of autotomy would not have been detected in this survey. Mutants with severe defects in the process of molting are unable to progress beyond the first or second larval stage, and therefore could not be tested. The 60-odd mutants that have been examined are discussed below under several headings, though there is much overlap in cases such as those affecting multifunctional kinases.

First, morphological mutants such as those with abnormal body shape or altered surface properties were considered. Severely shortened worms failed to form worm-stars and therefore could not be tested but several dumpy or small mutants (*dpy-12*, *dpy-17*, *sma-2*, *dbl-1*) were seen to be capable of autotomy. Long mutants (*lon-1*, *lon-2*, *lon-3*) did not exhibit any enhancement of autotomy. One mutant that has a larval roller phenotype as a result of its helically twisted body, the dominant *rol-6* mutant, was able to autotomize. Many mutants with cuticular surface alterations are hypersensitive to Verde1 [3,13] and therefore could not be tested, but *bus-10* mutants [3,14] were found competent to autotomize.

Second, mutants with known or potential alterations in cell death programs [15] were examined, in view of their possible defects in tissue remodeling. No effects on autotomy were seen in mutants defective in the main cell death pathway (*ced-3*, *ced-4*, *ced-9(gf)*, *egl-1*, *ced-13*), or in either of the corpse engulfment pathways (*ced-1*, *ced-2*, *ced-6*, *ced-7*, *ced-8*, *ced-12*, *mtm-1*, *psr-1*), or in nuclear breakdown (*nuc-1*), or in alternative caspases (*csp-1*, *csp-2*, *csp-3*).

Third, studies of neuronal regeneration and damage response have implicated many genes in these processes [16,17,18]. None of the regeneration mutants tested (*dlk-1*, *mak-2*, *mkk-4*, *pmk-3*) were found to be defective in autotomy. The genes *dapk-1*, *efa-6*, *jnk-1* and *kgb-2* act as negative regulators of regeneration, but mutants in these genes did not exhibit any noticeable enhancement of autotomy. Also, mutants in a few genes that contribute to cell migration or neuronal migration (*sdn-1*, *lim-9*, *mig-1*, *max-2*, *nlg-1*, *wrk-1*) were tested and found competent to autotomize.

Fourth, mutants altered in responses to pathogenic damage or defective in innate immunity [19] (*nsy-1*, *pik-1*, *pmk-1*, *dbl-1*, *jkk-1*, *dcar-1*) or to various kinds of stress (*hsf-1*, *sod-1*, *hif-1*, *egl-9*, *rict-1*, *daf-16*) were found competent to autotomize.

Lastly, a few mutants defective in developmental signaling, differentiation or molting (*ceh-10*, *cwp-4*, *fbn-1*, *lin-2*, *ksr-1*, *lin-42*, *mpk-1*, *ptr-23*, *rbr-2*, *sli-1*) were tested, again without observing any noticeable defects in autotomy.

## 4. Discussion

The survey of rhabditid species described in this report shows that the ability to undergo autotomy at the L4-to-adult molt is not confined to *C. elegans*, and may be widespread among small nematode species. All of the tested Caenorhabditis species and wild isolates of *C. elegans* that were susceptible to forming larval worm-stars after exposure to Verde1 bacteria in liquid also exhibited some autotomy. This process did not depend on continuing exposure to the pathogenic bacteria, only on the formation of stably knotted larval worms. It is possible that, in their natural environments, worms may become entrapped by their tails by pathogens other than Leucobacter Verde1 [3,20], such as nematophagous fungi [21,22], or by other naturally occurring adhesive factors, so the ability to escape entrapment by autotomy may be generally advantageous [20]. The observation that *C. elegans* can escape from L3 worm-stars by autotomy and sometimes still go on to produce self-progeny in adulthood indicates that autotomy can provide an escape mechanism throughout larval life. It may occur more frequently in the natural environment of this species than is apparent in laboratory culture.

In principle, it should be possible to test for autotomy in many nematodes species by using micromanipulation or adhesion to create artificial worm-stars. This would allow tests for autotomy to be applied to nematode species that are either too resistant or too hypersensitive to Verde1, and to *C. elegans* mutants that are too uncoordinated to form worm-stars. Formation of mixed worm-stars, as described previously [3], may also allow testing of additional strains and mutants.

The observation of twisted links between segments of incompletely autotomized worms revealed a plausible mechanical explanation for autotomy, whereby twisting squeezes the soft body of the worm down to a point where segments can be separated by pinching off. The lethargus-associated flipping that precedes molting [11] was found to be unnecessary for autotomy, but twisting movements can still occur in flipping-defective mutants. Twisting may provide a means of severing body parts for other soft-bodied invertebrates.

Successful autotomy must also require substantial tissue healing at the site of scission. Both epidermal and intestinal tissues appear to undergo fusion or adhesion, creating a tightly sealed posterior end, but adequate details of how this occurs, and how other tissues respond, would require extensive analysis by electron microscopy. Examination using tissue-specific fluorescent reporters has not been illuminating so far.

The healing associated with autotomy seems likely to occur as a modification or side effect of the normal process of molting. Molting has been less thoroughly investigated than many other aspects of *C. elegans* growth and development, despite its central importance to nematode biology [10]. Using an RNAi screen, Frand et al. identified 159 genes that are required for molting, most of which remain to be investigated in detail [23]. Mutants of many of the molting-associated genes that have been identified in this and other screens [23,24,25,26] result in variable or impenetrant defects in molting rather than complete failure, so these could be tested for specific defects in autotomy, but at present it is difficult to predict which might be the most informative candidates to test. One mutant affecting such a gene, *ptr-23*, was tested and found to be capable of autotomy, despite its known defects in molting [24]. A weak mutant of the fibrillin-related gene *fbn-1*, which is required for molting [25] was also found to be capable of autotomy. Testing other molting gene candidates by means of RNAi knockdown has not been attempted in view of the variability in knockdown efficiency and the lack of a quantitative assay for autotomy.

No detectable major regeneration of lost body parts was seen in either L4 or adult autotomized worms. This is not surprising, in view of the largely invariant cell lineage and development of *C. elegans* and other nematodes, and the fact that most somatic cells in late larvae and adults are post-mitotic. However, plasticity at the level of wound healing and axonal regeneration does occur in *C. elegans*, and has been much studied [16,17,18]. Many genes involved in these processes have now been identified, but none of the corresponding mutants that were tested in this initial survey were found to be incapable of autotomy.

Autotomy represents a major trauma, so some of the pathways activated in response to pathogenic, physical or chemical assault might be important during and after autotomy. However, mutants defective in a variety of these pathways were still able to autotomize.

A general caveat to these conclusions and the failure to identify any genes essential for autotomy is that delays or inefficiency in autotomy, or reduced survival of truncated worms, would not have been easily detected. Nevertheless, the severing and healing processes appear to be robust and largely independent of known developmental pathways, and may involve previously unidentified cellular and molecular mechanisms. One possible way to identify a specific autotomy program might be to search directly for autotomy-defective mutants. This could be done by means of a clonal mutagenesis screen. In view of the reliability and robustness of the autotomy process, such a screen should be feasible.
